# In search of nano-materials with enhanced secondary electron emission for radiation detectors

**DOI:** 10.1038/s41598-021-89990-y

**Published:** 2021-05-18

**Authors:** Marian Cholewa, Mario Cappellazzo, Mario Ley, Dennis Bittner, Jan Jolie, Keundong Lee, Minho Song, Gyu-Chul Yi, Plamen Boutachkov

**Affiliations:** 1 Institute of Physics, College of Natural Sciences, University of Rzeszow, Pigonia 1, 35-959, Rzeszow, Poland; 2 Institut Für Kernphysik, University of Cologne, Zülpicher Straße 77, 50937 Cologne, Germany; 3 Department of Physics and Astronomy, Seoul National University, Seoul, 151-747 Republic of Korea; 4 GSI Helmholtzzentrum Für Schwerionenforschung GmbH, Planckstraße 1, 64291 Darmstadt, Germany

**Keywords:** Materials science, Physics

## Abstract

There has been limited research devoted to secondary electron emission (SEE) from nano-materials using rapid and heavy ion bombardment. Here we report a comparison of SEE properties between novel nano-materials with a three-dimensional nano-structure composed of a mostly regular pattern of rods and gold used as a standard material for SEE under bombardment of heavy ions at energies of a few MeV/nucleon. The nano-structured materials show enhanced SEE properties when compared with gold. Results from this work will enable the development of new radiation detectors for science and industry.

## Introduction

Electrons created in the interaction of charged particles and radiation with matter can escape from the material surface. This phenomenon is called Secondary Electron Emission (SEE) and is utilized in particle and radiation detection. The secondary electron yield is determined by the bulk and surface properties of the material^1,2^. Nano-structures and nano-materials may significantly improve the SEE yield through the manipulation of material geometry and surface properties. A large amount of work has been presented on the field emission of nano-materials^3–13^ whilst little is known about their effect on SEE^14,15^ properties.

This paper presents an investigation on the influence of ZnO nanorods and GaN coating, a high-band material, on the observed SEE from heavy ions. The study was performed with ^73^Ge-beam and ^16^O-beam with energies of 1.4 MeV/u and 2.5 MeV/u, respectively. A higher SEE yield was observed for the investigated nano-materials compared to the Au foil.

During recent decades, some scientific research^16–22^ has been focused on material properties for obtaining anti-multipactor coatings of low SEE. The community of a high energy physics and the European Space Agency (ESA) is leading a technological research on a new approach based on surface roughness that might perform as a kind of blackbody or Faraday cage effect.

A community of high energy physics^16,17^ has been working on development of efficient techniques to reduce the SEE (secondary electron yield—SEY or δ) from elements of beam line developed from stainless steel, copper or aluminium. In works^16,17^ authors have shown reduction of the SEE by introducing micro and nanostructures to the surface of stainless steel, copper or aluminium by a nanosecond pulsed laser irradiation. The SEE of modified metal surfaces are being reduced. A simplified 2D theoretical model^18^ has been applied to calculations of the molecular pumping properties of complex vacuum systems. The suppression efficiency of the SEE from Cu as a function of roughness parameter has been presented.

A multilayer coating structure^19^ was adopted for fulfilling the stringent requirements of the space. The surface of a standard silver plating was modified by a two-step treatment. This nanostructure was efficient in reducing the SEE properties of the surface. Another group^20^ performed numerical simulations on complex structures called velvet structures (vertically standing whiskers) and developed an approximate analytical model that calculates the net secondary SEE yield from a velvet surface as a function of the velvet whisker length and packing density, and the angle of incidence of primary electrons. The reduction in the SEE occurs due to the capture of low-energy, true secondary electrons emitted at the bottom of the structure and on the sides of the velvet whiskers. Among total SEE suppression techniques, micro-porous surfaces have been demonstrated as an effective method. In publication^21^ authors developed an analytical model that is able to obtain the contributions of total SEE from both the 1st and 2nd generation secondary electrons. Complex structures on a material surface can significantly reduce the total SEE from that surface. The reduction occurs due to the capture of low-energy, true secondary electrons emitted at one point of the structure and intersecting another. The authors^22^ performed Monte Carlo calculations to demonstrate that fractal surfaces can reduce net SEE produced by the surface as compared to the flat surface.

Works^16–22^ indicated different approach about SEE properties of micro and nanomaterials developed at different surfaces. In these works authors are trying to develop structures that will reduce SEE properties and for this task they have been developing theoretical models and perform experiments that will prove their point of view. Authors are trying to develop suppressing layers on different materials including stainless steel, coper and aluminum. In case of our research^14,15^ we are trying to develop and investigate nanostructures made of different nanomaterials, including ZnO and ZnO/GaN that will show increase in the SEE properties. Both directions are not excluding each other, but supplementing each other and gave full picture in relations to nanomaterials.

In this article we did not compare the performance of modyfied surface with its flat and unmodified counterpart. However, this work was performed in reference^14^ where performance of ZnO, ZnO/GaN and ZnO/AlN nanostructures were compared with flat ZnO, AlN and GaN. In this work nanostructured samples performed much better than flat samples. It was decided not to repeat previous works but perform comparison with Au flat surface only.

After some research of the SEE properties of selected nanomaterials we still could not answer the detailed question: is this enhancement in SEE properties a function of nanostructure only or some other factors play important role? We are planning more detailed works on this topic in the future.

## Main part

### Motivation

The role of secondary electrons in modern science and industry was recently outlined in-depth by Trucchi et al.^23^. These detectors have various applications in basic research and industry practices. Nano-structures and nano-materials may significantly improve the performance of these detectors by providing a higher SEE^14,15^. Cholewa^14,15^ et al. in collaboration with the National University of Singapore (NUS), initiated an investigation of the SEE properties of various nano-materials. Very promising results were obtained, leading to a patent in the USA and publication^14^. Our recent work^15^ conducted at the GSI laboratory confirmed advantage of using 1D (one dimensional) nanomaterials to obtain enhancement of the SEE properties. A large amount of work has been conducted for the development and characterization of physical properties of carbon nanotubes^24^ and nanorods^25^, as well as their field emission capabilities. This research has mostly been driven by the need for the development of a new generation of flat-screen displays. However, there has been little research of secondary electron detectors based on nano-materials. The pioneering studies of ZnO nano-rods by Cholewa^14,15^ et al. indicated enhanced SEE, pointing to the above materials as being a potential candidates for the development of new detectors for ionizing radiation. The purpose of this study is to re-establish the production technology of the materials investigated by Cholewa^14,15^ et al. and to further explore their response to heavy ions.

### Set-up

The SEE of these materials can be characterized using a chevron stack of micro channel plates (MCPs) as an electron multiplier^26,27^. The pulse height distribution (i.e., the distribution of amplitude heights) of MCPs is a measure for the quantity of secondary electrons yield, despite the fact that the electron multiplication in the MCP stack is statistically distributed. The higher the pulse height, the higher the expectation value for the number of secondaries which produce the observed signal. Electron multiplication in MCPs, i.e., their gain, depends exponentially on the applied bias voltage and changes with age. Therefore, care must be taken to control the gain of the MCP so as not to lose comparability.

**Figure 1:**
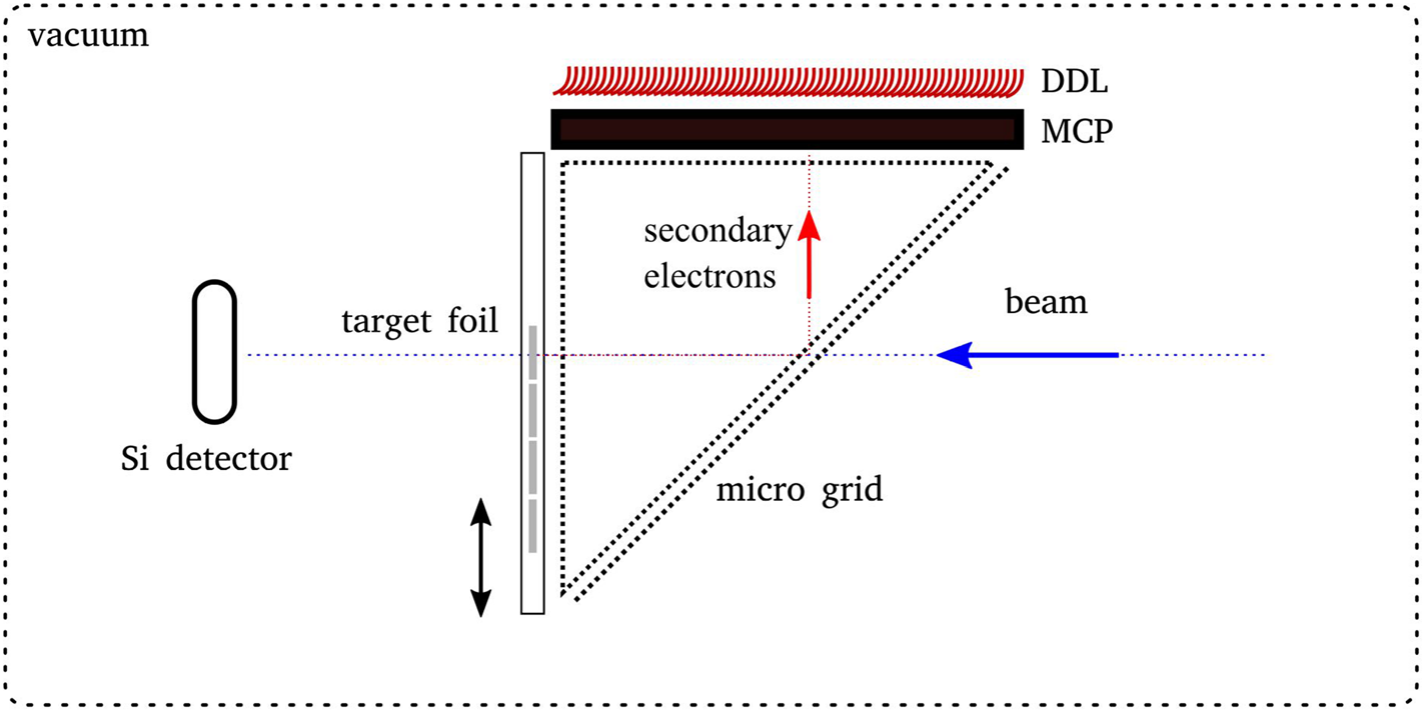
This figure shows a schematic of the experimental set-up for testing the SEE of thin material samples. Secondary electrons generated by ionizing radiation are detected with a micro-channel-plate (MCP) detector connected to a CFD1x from *RoentDek* for pulse height measurement. Secondary electrons are guided to the MCP via an electrostatic imaging system. Single ions are detected with an Si detector located behind the target.

A schematic drawing of the experiment set-up is presented in Fig. 1. Collimated ionizing radiation (heavy ions in our case) enter the set-up and release secondaries of a sample. Produced secondaries are guided to the MCP via an electrostatic imaging system consisting of an electrostatic mirror and a grounded field cage^26^. The sample itself is biased at ~  − 2 kV to collect most of the secondaries and to ensure that these secondaries can reach the MCP surface which is biased >|− 1.3 kV|. A dual delay line^28^ (DDL) anode behind the chevron stack allows a position-sensitive measurement. Extremely thin samples were chosen so that the used ion beams could penetrate them. A silicon surface barrier detector (Si detector) was installed ~ 30 cm behind the target foils to characterize the SEE coincident to the passing ions and distinguish events of spontaneous electron emission from the foil and dark counts from the MCP. The entire set-up was under a high vacuum in the order of 1–2 × 10^−6^ mbar. To enable a convenient exchange of multiple samples, without opening the vacuum chamber, a target ladder with five positions was designed. It was also possible to rotate the set-up by 180° to study the SEE, either in a backward or forward hemisphere, respective to the beam. Type ZX60-33-LN-S + from *Mini-Circuits* was used for further amplification of the signal coming from the MCP. The pulse height distribution was measured with a CFD1x from *RoentDek*.

### Samples

**Figure 2:**
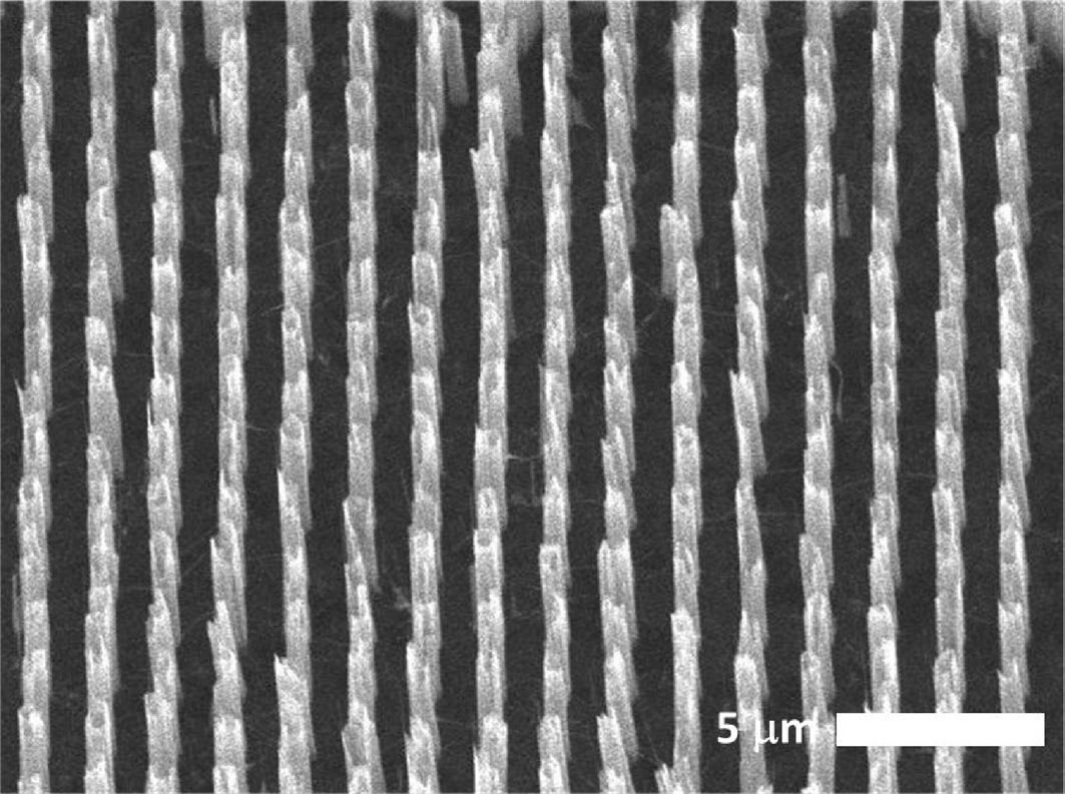
Scanning Electron Microscope (SEM) image displays the ZnO nano-rods. The rods have a length of 5 µm and are separated by 1 µm.

For sample substrates we used thin (< 1 µm) silicon nitride (Si_3_N_4_) foils. On these foils classic materials such as aluminum or gold, currently used for SEE, were evaporated as ultra-thin layers (< 40 nm). The nano-structures were also grown on these foils. To enhance the electric conductivity for a more homogeneous electric field of the applied bias voltage, and for growing the nano-structures, graphene films were added to the first step via Chemical Vapor Deposition (CVD). Then, position-controlled ZnO structures were grown via Metal Organic Vapor Phase Epitaxy (MOVPE) as described by Park et al.^29^ In an optional subsequent step, the ZnO nano-rods were covered with GaN coating. The nano-rods had a length of ~ 5 µm and a thickness of ~ 1 µm, with a pitch distance of ~ 1 µm (cf. Fig. 2). For each nano-material we measured two samples with different sample sizes.

## Results

Integral, mean and root mean squared of the measured distributions for the 100 MeV ^73^Ge beam. For non-saturation a cut below the 1220 channel was used. N.B. the forward and backward sections were not measured with the same MCP gain.

**Figure 3:**
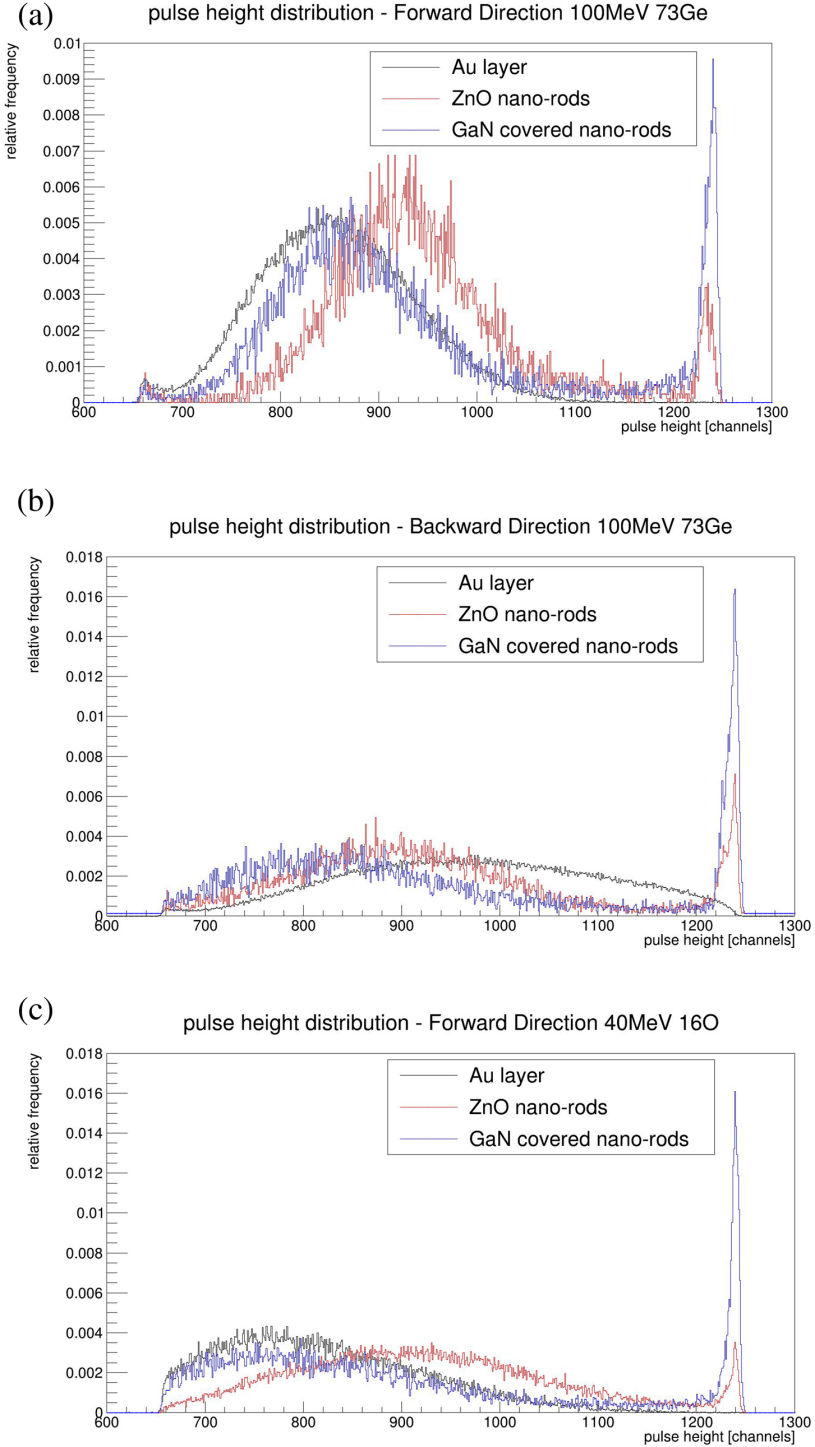
Pulse height distribution for gold and the nano-structured materials under bombardment of ^73^Ge at ~ 1.4 MeV/u. In (**a**) the SEE in the forward direction is shown with an MCP bias at − 1550 V. In (**b**) it is shown in a backward direction with a bias of − 1650 V. SEE in the forward direction is shown with a ^16^O-beam at 2.5 MeV/u in (**c**). MCP bias was set to − 1700 V in (**c**).

Table 1 shows the results of the measurement of the samples for the ^73^Ge beam measurement. Additionally, in Fig. 3 some of the measured pulse height distributions are shown. MCP gain was adjusted in such a way that it had a ~ 100% detection efficiency for the Au sample, and quasi-no events in saturation. Here, detection efficiency indicated the ratio between detected ions by the secondary electron detector and detected ions by the Si detector. This was achieved for SEE under forward angles with a bias voltage of − 1550 V. The resulting pulse height distribution related to the SEE, as described above for the forward SEE of gold, is shown in Fig. 3a as a black curve. This curve can be fairly described with a Gaussian distribution with a mean of 856 channels and a standard deviation of 77 channels. Keeping the gain, but switching to ZnO nanorods, the distribution which is shown in red in Fig. 3a is no longer well-described by Gaussian distribution alone. It seems to have an approximately Gaussian part at low SEE and a long tail towards higher yields. Events with very high yields were summed up at saturation peak of around channel 1230. The mean of the approximately Gaussian part has shifted to higher yields compared to Au. In saturation are nearly 3% of all events. Compared to this, the blue curve of GaN covered nano-rods in Fig. 3a show a lower mean value of the peak at lower yields, yet are still higher than the absolute mean of gold. But nearly 13% of all events are in saturation. From this we can conclude that the real mean value should be higher for GaN covered nano-rods than for bare ZnO ones. By decreasing the MCP gain, one can look inside the distribution, which is hidden inside the saturation peak at higher gains. Here it can be found that for GaN there is no further structure, but rather a long tail towards higher yields.

**Table 1:** Summarized results from the experiment with 100 MeV ^73^Ge ions.

Sample	Total			Non-saturation			Saturation
	Integral	Mean (chan.)	RMS (chan.)	Integral	Mean (chan.)	RMS (chan.)	Integral
**Forward 100 MeV **^**73**^**Ge**							
Au	0.9997	857.9	78.4	0.9995	857.9	78.2	0.0002
ZnO large	0.9953	928.3	120.2	0.9692	920.1	110.7	0.0257
ZnO small	0.9972	945.6	103.1	0.9525	932.2	83.9	0.0444
ZnO/GaN medium	0.9996	935.1	149.0	0.8729	891.3	101.4	0.1258
ZnO/GaN small	0.9997	926.5	144.7	0.8887	888.0	100.7	0.1104
**Backward 100 MeV **^**73**^**Ge**							
Au	0.9991	971.3	128.1	0.9878	968.4	125.8	0.0104
ZnO large	0.9955	985.6	137.2	0.9079	961.4	118.3	0.0863
ZnO small	0.9939	930.8	144.8	0.9046	900.9	114.3	0.0880
ZnO/GaN medium	0.9934	945.0	186.2	0.7838	867.3	123.9	0.2072
ZnO/GaN small	0.9929	899.9	184.3	0.8238	831.0	114.3	0.1682

As expected, for the backward direction the yield was found to be lower. Hence, we had to increase the MCP gain by decreasing the bias voltage to − 1650 V. These results are shown in Fig. 3b. The shape of the distributions of the nano-materials seemed unchanged, but the distribution of gold is no longer described effectively by a Gaussian. Furthermore, its mean became higher than the mean of the low yield peak of both nano-materials. With this gain ~ 9% of the events coming from ZnO and ~ 20% from GaN were in saturation. Only 1% of the events of gold were near the saturation limit.

In Fig. 3c a measurement with a ^16^O-beam was performed. Since the energy loss was lower that of the ^73^Ge-beam we expected the observed behavior of a lower SEE, yet the shape of the distributions hardly changed. We observed a cut-off in gold, despite the increased MCP gain. Hence, gold only has a detection efficiency of 96.6% in this configuration. In addition, the mean value of gold was below the mean of both of the other distributions.

## Discussion

The results of the two samples types do not differ as significantly as expected. We can conclude that the new nano-structured materials show a higher secondary electron yield compared to gold, at least for emissions in the forward direction. This can be seen in the mean value of the total distribution in Table 1. For backward emissions, the interpretation is less straightforward, as up to 20% of the events concluded in the saturation peak. Hence, their real pulse height is unknown. From the observation that the structure appears as a long tail that is basically equally distributed, their real mean is expected to be higher than the mean of gold. The distribution of the nano-structured material seems to be composed of a “Gaussian” part or peak, and a long tail.

**Figure 4:**
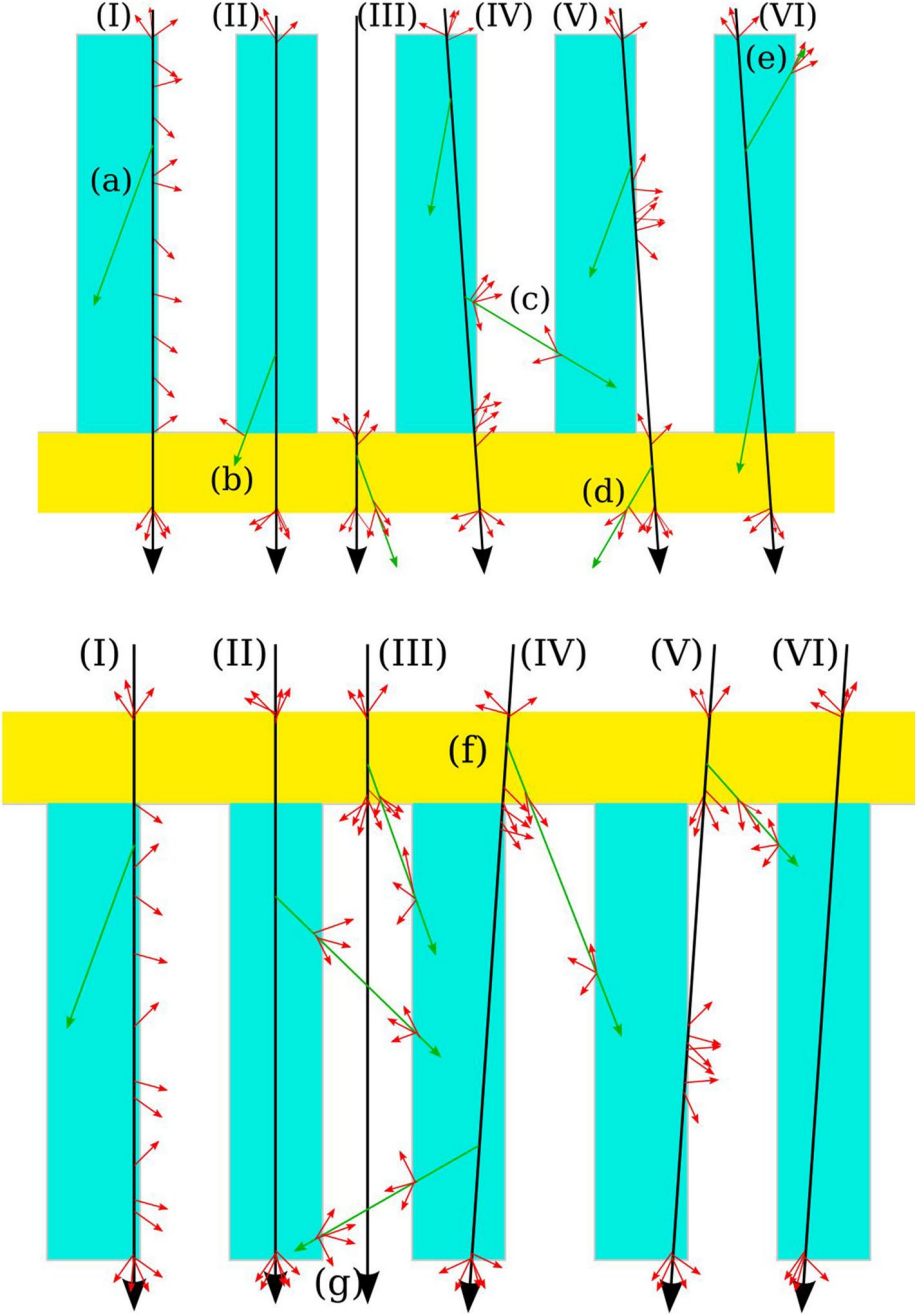
(**a**) Schematic drawing of possible interactions with ions (black) with sample (I–VI). The maximum angle with which ions could have passed through the sample is below 4°. Here, emission in a backward direction is shown. Emission of slow electrons (red) can only occur near the surface. Moreover, delta-electrons (green) can cause emission of the slow electrons near the surface (**a**–**e**). (**b**) Same as (**a**) but emission in forward direction is shown. Essentially, its difference to (**a**) is that delta-electrons from the window can hit the rods. This is also the case with (**f**), and mostly in forward directions emitted delta-electrons come closer to the end of the rods (**g**).

The difference in shape of the forward and backward electron yield distribution of the nano-materials could be due to the following mechanism: SEE is normally explained in three steps^2^ (production, transportation and emission). Though through the nano-structure transportation losses could be minimized, and through the coating emission, be facilitated. The long tail could indicate ions hitting a rod near the edges (cf. Fig. 4 I, II, IV, V), and the peak from ions which missed them or only passed just inside (cf. Fig. 4 III, VI). The difference in observation between forward and backward directions might be explained by the contribution of fast electrons. When ions pass through matter slowly (energy < 10 eV) and rapidly (> > 10 eV) secondary electrons are produced with different transportation lengths^1^. Rapid secondary electrons can cause the production of additional (slow) secondary electrons. Their creation complicates the picture (cf. Fig. 4a–g). They are mainly produced in a forward direction^1^. Since their transportation length is longer than the length of slow secondaries, they could benefit more from the nano-structures. Their contribution is normally used to explain the difference between the forward and backward secondary electron yields of thin foils. This anisotropy could be the dominant part of the explanation of the measured difference. Considering the nano-structure the main difference seems to be that in forward direction additional delta-electrons can arrive from the window (cf. Fig. 4f,g). For delta-electrons produced inside the rods, not too close to the window, it is difficult to conceive of difference of their contribution to the yield. Thus far we cannot conclusively state if there is a difference in the tails for the forward and backward directions, and the importance of delta-electrons. It is still possible that there is almost (cf. Fig. 4f) no difference for the tail and that the (expected) shift in the “Gaussian part” could be explained by the delta-electrons, as known from the existing literature^6^. On the other hand, a difference could arise through the higher production of secondaries near the end of the rods in forward directions. Secondaries produced near the end should have a higher escape probability from the nanorod forest, and possibly see a higher extraction field. Unfortunately, the electrical extraction field in the forest is yet unknown.

Ultimately, we have re-established the production technology of the materials reported in publications^14,15^. We used a ^73^Ge-beam and ^16^O-beam with energies of 1.4 MeV/u and 2.5 MeV/u, respectively, to study the SEE properties of the above materials. We observed enhanced secondary electron yields, compared to gold, in both forward and backward directions. However, the very basic properties of these nano-materials for production, and secondary electron emissions, are still unknown and require further study. In future, different measurement techniques allowing for higher electron yields should be used. The sizes and different shapes of nano-structures have to be studied experimentally within proper simulations, and different coatings also need to be examined.

All experimental techniques used in works^14,15^ are different to avoid possible systematic errors associated with single technique.

The fact the SEE properties for nanomaterials do differ in forward and backward direction and this could be related to the fact that in each experiment nanostructure of the sample is facing the MCP detector. The set-up voltages are different for the MCP detector. This effect has been investigated in detail in the past by Rothard^30^ et al. Our data also indicated that SEE yield is higher in forward direction.

In our works we used beams with current below 10 fA and the beam was large. Such current and beam density is to low to cause any damage to the sample. We have obserwed this fact by using the same sample over the long period of time for more that 10 years in different experiments at different facilities. Experiments with larger currents in the order of pA or nA will be able to answer the question about sample stability and resistance to radiation damage.

## Data Availability

The data that support the findings of this study are primarily available within the paper. Additional data is available upon request from the corresponding author.

## References

[1] Rothard H, Jung M, Caron M, Grandin J-P, Gervais B, Billebaud A, Clouvas A, Wünsch R (1998). Strong projectile-dependent forward-backward asymmetry of electron ejection by swift heavy ions in solids. Phys. Rev. A.

[2] Schou J (1980). Transport theory for kinetic emission of secondary electrons from solids. Phys. Rev. B.

[3] Hasselkamp D, Rothard H, Groenveld K-O, Kwmmler J, Varga P, Winter H (1992). Particle Induced Electron Emission II.

[4] Ning-Sheng Xu, Deng S-Z, Chen J (2003). Nanomaterials for field electron emission: preparation, characterization and application. Ultramicroscopy.

[5] Zhu YW, Yu T, Cheong FC, Xu XJ, Lim CT, Tan VBC, Thong JTL, Sow CH (2004). Large-scale synthesis and field emission properties of vertically oriented CuO nanowire films. Nanotechnology.

[6] Zhong Lin Wang (2008). Splendid one-dimensional nanostructures of zinc oxide: a new nanomaterial family for nanotechnology. ACS Nano.

[7] Zeng H, Xu X, Bando Y, Gautam UK, Zhai T, Fang X, Liu B, Golberg D (2009). Template deformation-tailored ZnO nanorod/nanowire arrays: full growth control and optimization of field-emission. Adv Funct Mater.

[8] Saito Y (2010). Carbon Nanotube and Related Field Emitters: Fundamentals and Applications.

[9] Palnitkar UA, Kashid RV, More MA, Joag DS, Panchakarla LS, Rao CNR (2010). Remarkably low turn-on field emission in undoped, nitrogen-doped, and boron-doped graphene. Appl. Phys. Lett..

[10] Li G, Li Y, Qian X, Liu H, Lin H, Chen N, Li Y (2011). Construction of tubular molecule aggregations of graphdiyne for highly efficient field emission. J. Phys. Chem. C.

[11] Zhai T, Li L, Liao YMM, Wang X, Fang X, Yao J, Bandoa Y, Golberg D (2011). One-dimensional inorganic nanostructures: synthesis, field-emission and photodetection. Chem. Soc. Rev..

[12] Zou R, He G, Xu K, Liu Q, Zhang Z, Hu J (2013). ZnO nanorods on reduced graphene sheets with excellent field emission, gas sensor and photocatalytic properties. J. Mater. Chem. A.

[13] Mahmood K, Park SB, Sung HJ (2013). Enhanced photoluminescence, Raman spectra and field-emission behavior of indium-doped ZnO nanostructures. J. Mater. Chem. C.

[14] Cholewa M, Moser HO, Huang L, Lau SP, Yoo J, An SJ, Yi G-C, Xingyu G, Wee ATS, Bettiol A, Watt F, Fischer B (2007). Secondary electron emission properties of III-nitride/ZnO coaxial heterostructures under ion and X-ray bombardement. Nucl. Instrum. Methods B.

[15] Boutachkov P, Voss KO, Lee K, Song MS, Yi C, Cappellazzo M, Kondziołka W, Liskowicz A, Cholewa M (2021). An investigation of secondary electron emission from ZnO based nanomaterials for future applications in radiation detectors. Sci. Rep..

[16] Valizadeh R, Malyshev OB, Wang S, Zolotovskaya SA, Gillespie WA, Abdolvand A (2014). Low secondary electron yield engineered surface for electron cloud mitigation. Appl. Phys. Lett..

[17] Valizadeh R, Malyshev O, Wang S, Sian T, Cropper MD, Sykes N (2017). Reduction of secondary electron yield for E-cloud mitigation by laser ablation surface engineering. Appl. Surf. Sci..

[18] Krasnov A (2004). Molecular pumping properties of the LHC arc beam pipe and effective secondary electron emission from Cu surface with artificial roughness. Vacuum.

[19] Nistor V, González LA, Aguilera L, Montero I, Galán L, Wochner U, Raboso D (2014). Multipactor suppression by micro-structured gold/silver coatings for space applications. Appl. Surf. Sci..

[20] Swanson C, Kaganovich ID (2016). Modeling of reduced effective secondary electron emission yield from a velvet surface. J. Appl. Phys..

[21] Ye M, Wang D, He Y (2017). Mechanism of total electron emission yield reduction using a micro-porous Surface. J. Appl. Phys..

[22] Swanson C, Kaganovich ID (2017). “Feathered” fractal surfaces to minimize secondary electron emission for a wide range of incident angles. J. Appl. Phys..

[23] Trucchi DM, Melosh NA (2017). Electron-emission materials: advances, applications, and models. MRS Bull..

[24] Thong JTL, Oon CH, Eng WK, Zhang WD, Gan ML (2001). MoS_2_ nanoflowers and their field-emission properties. Appl. Phys. Lett..

[25] Li YB, Bando Y, Golberg D (2004). ZnO nanoneedles with tip surface perturbations: excellent field emitters. Appl. Phys. Lett..

[26] Liénard E, Herbane M, Ban G, Darius G, Delahaye P, Durand D, Fléchard X, Labalme M, Mauger F, Mery A, Naviliat-Cuncic O, Rodríguez D (2005). Performance of a micro-channel plates position sensitive detector. Nucl. Instrum. Methods Phys. Res. Sect. A.

[27] Jagutzki O, Mergel V, Ullmann-Pfleger K, Spielberger L, Spillmann U, Dörner R, Schmidt-Böcking H (2002). A broad-application microchannel-plate detector system for advanced particle or photon detection tasks: large area imaging, precise multi-hit timing information and high detection rate. Nucl. Instrum. Methods Phys. Res. Sect. A.

[28] Kozulin EM, Bogachev AA, Itkis MG, Itkis IM, Knyazheva GN, Kondratiev NA, Krupa Ľ, Pokrovsky IV, Prokhorova EV (2008). The CORSET time-of-flight spectrometer for measuring binary products of nuclear reactions. Instrum. Exp. Tech..

[29] Park JB, Oh H, Park J, Kim N-J, Yoon H, Yi G-C (2016). Scalable ZnO nanotube arrays grown on CVD-graphene films. APL Mater..

[30] Rothard H, Caraby C, Cassimi A, Gervais B, Grandin J-P, Jardin P, Jung M (1995). Target-thickness-dependent electron emission from carbon foils bombarded with swift highly charged heavy ions. Phys. Rev. A.

